# The early history of the ESID registry

**DOI:** 10.70962/jhi.20250248

**Published:** 2026-02-18

**Authors:** Roberto Paganelli

**Affiliations:** 1 UniCamillus International Medical University Rome, Italy; 2 SYNERGO, Pescara, Italy

## Abstract

The history of the ESID registry is described with the help of original documents of the precursor EGID going back to 1994.

The extremely interesting article by Gerhard Kindle et al. (1) reporting data from the registry of the European Society for Immunodeficiencies (ESID-R) briefly mentions in the first paragraph that the ESID-R was established as a hard copy–based database in 1994 (1), and this offers me the occasion to describe more extensively the origin of the registry, whose history has not been recorded yet in full.

The European Group for Immunodeficiencies (EGID), precursor of ESID, established the EGID registry in January 1994, as part of a concerted action operating with a grant from the European Union (E.U.) (see below). The EGID had been founded in 1983 at a meeting organized in Rome (Italy) by Prof. F. Aiuti (2), and it organized six biennial meetings until, at the sixth EGID biennial meeting held in Sitges (Spain) in October 1994, it was decided for EGID to officially become ESID. By 1983, reports of two national registries from Europe had already been published (3, 4), and the World Health Organization Scientific Group on Primary Immunodeficiencies had just published its first report (5).

At the fifth EGID meeting, held in Lugano (Switzerland) in 1992, a proposal for an E.U. concerted action on primary immunodeficiencies funded by the BIOMED-I was decided with Prof. A. Fischer as coordinator. The EGID president Prof. R. Seger informed all participants of the success of the application in a newsletter of the EGID in January 1994 (Figs. S1, S2, and S3). The first goal of the concerted action was the establishment of a European Registry of Primary Immunodeficiencies, and a Working Party composed of L. Hammarstrom (Huddinge, Sweden), G. Morgan (London, UK), and myself in Rome was appointed (Fig. S4). The data were provided on paper forms similar to those used by the existing national registries and transmitted by fax or mail to Huddinge, where they were stored in a dedicated computer.

**Figure S1.**
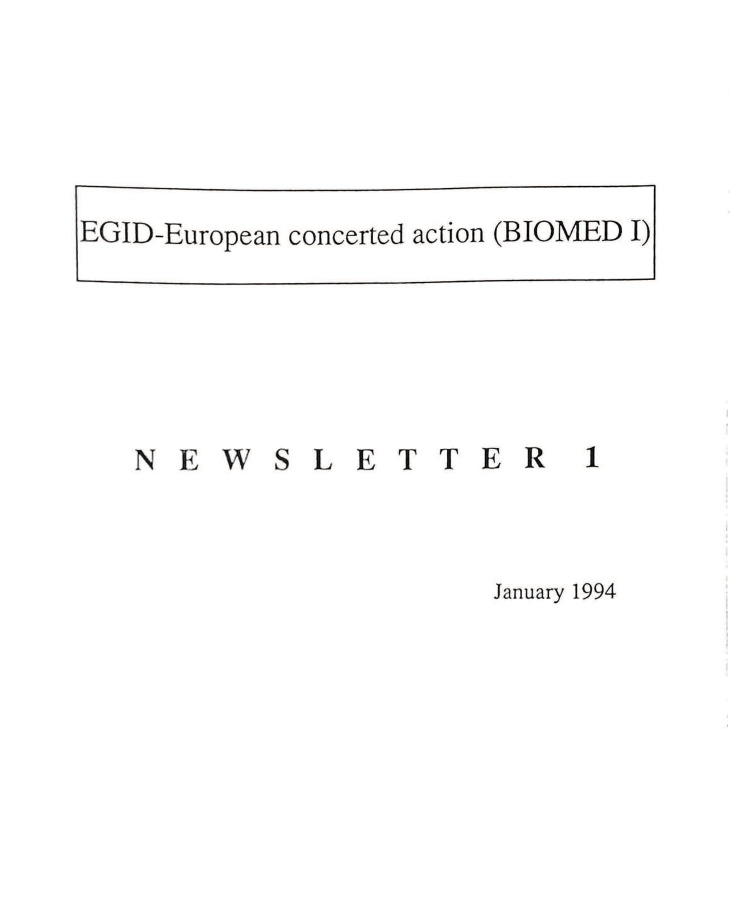
Front page of the newsletter of the EGID—concerted action (BIOMED-I).

**Figure S2.**
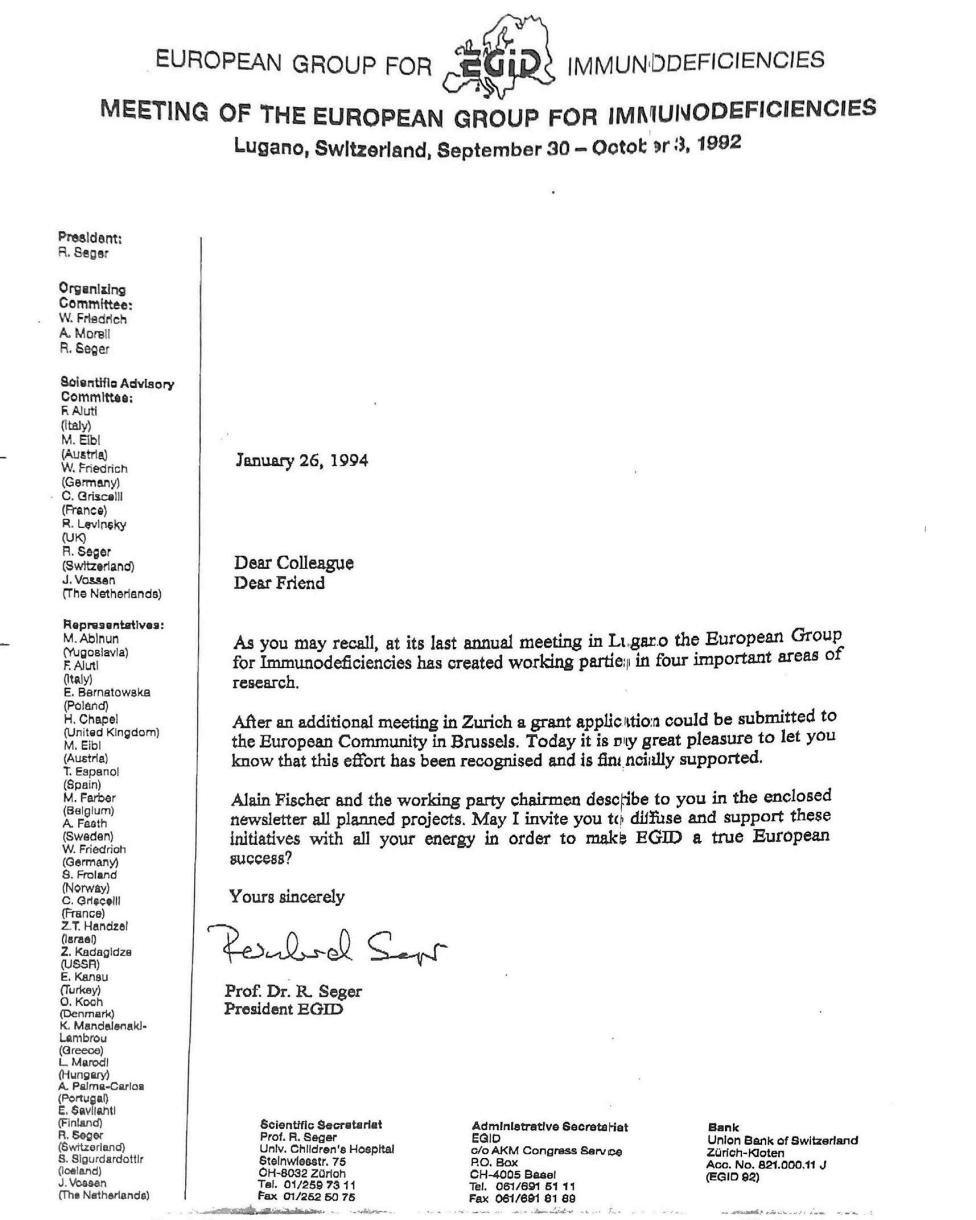
Letter from Prof. R. Seger announcing the success of the EGID application to the E.U.

**Figure S3.**
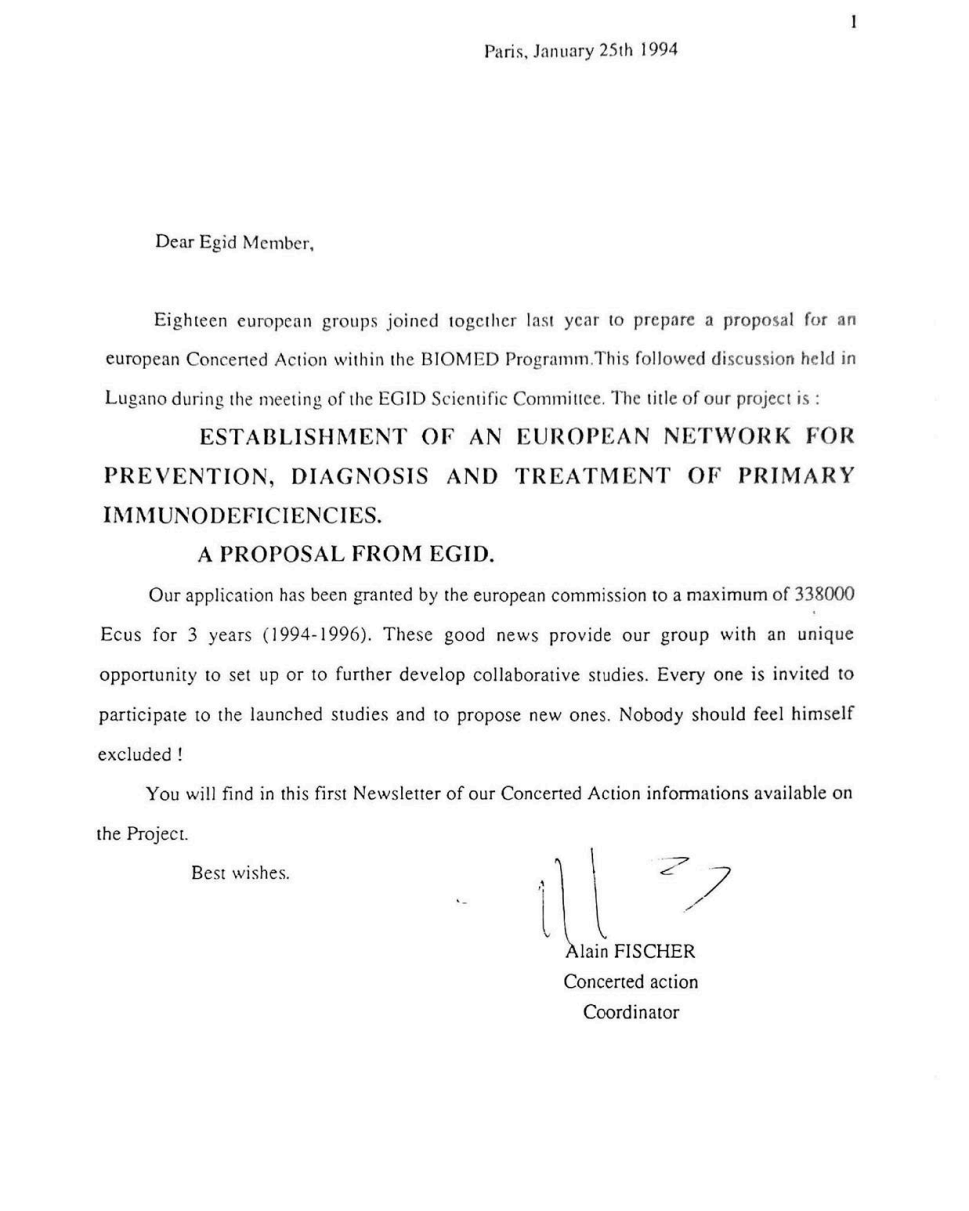
Letter from Prof. A. Fischer opening the newsletter of the concerted action.

**Figure S4.**
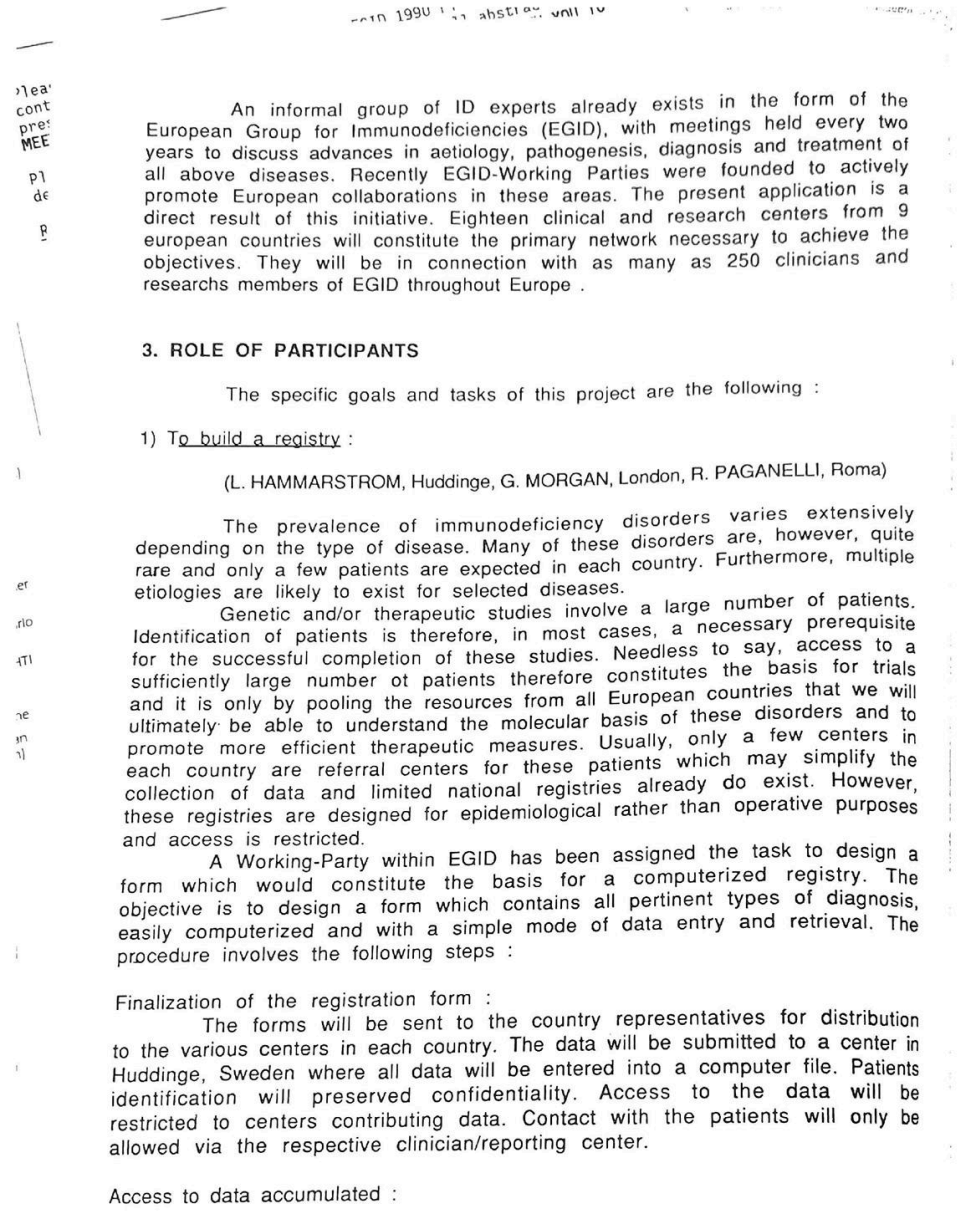
Goals and tasks of the EGID project with “1: To build a registry.”

The EGID Registry Working Party reported at the Sitges meeting that >500 patients had already been entered into the registry (Fig. 1, A and B). The primary center for the collection of data was in Huddinge, with additional centers in London and Rome. The following report of the now ESID-R, published in 1998 (6), included 7,616 cases from 25 countries, and a final one based on this registry reported 9,707 patients from 26 countries (7). Soon the registry was to change into an internet-based database (8), launched in June 2004, and stored on servers of the University of Freiburg, Germany. This led to the first report in 2007 of the new ESID-R (9) on 2,386 patients from 20 countries. The change from EGID-R to ESID-R was only in the name, so it is correct to date its establishment in 1994.

**Figure 1.**
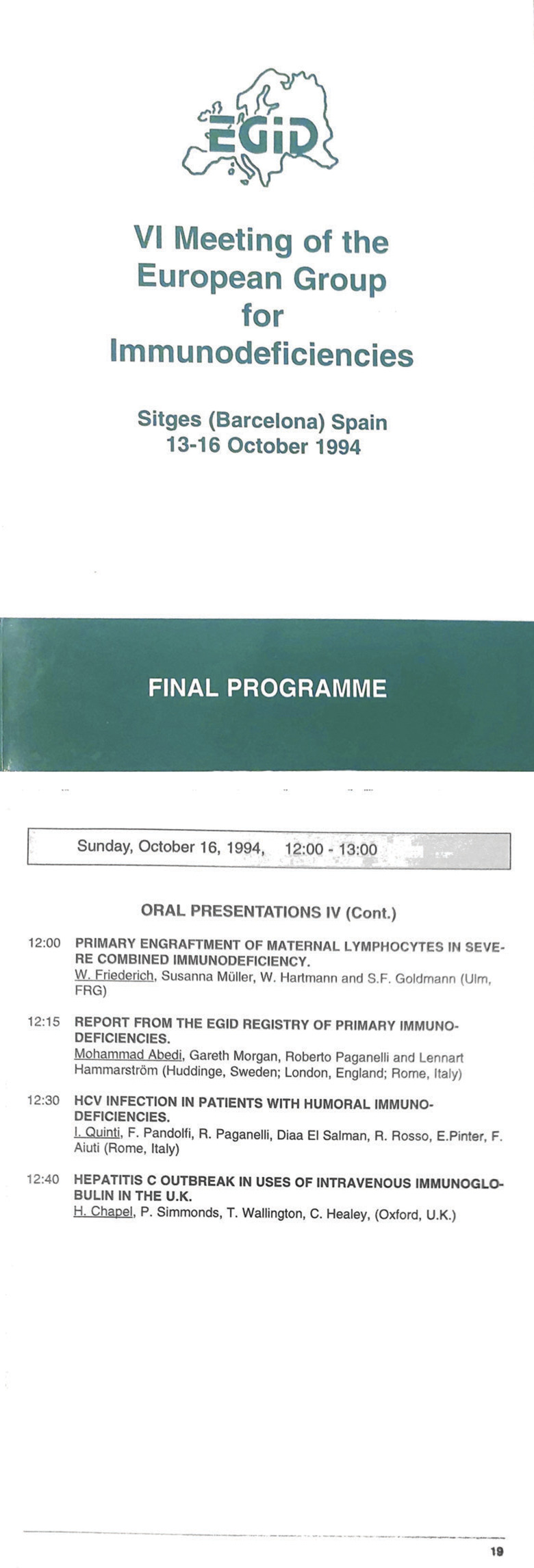
**The booklet with the congress program of the 6th EGID meeting (front page Top), and the page referring to the presentation of the first EGID Registry report.** Top: Front page of the program of the sixth EGID meeting in Sitges (1994). Bottom: Program page with the report of the EGID registry.

For anyone interested in the history of EGID, in Fig. S5, I reproduce the poster presented at the 21st biennial meeting of the ESID held in Marseille (France) in 2024.

**Figure S5.**
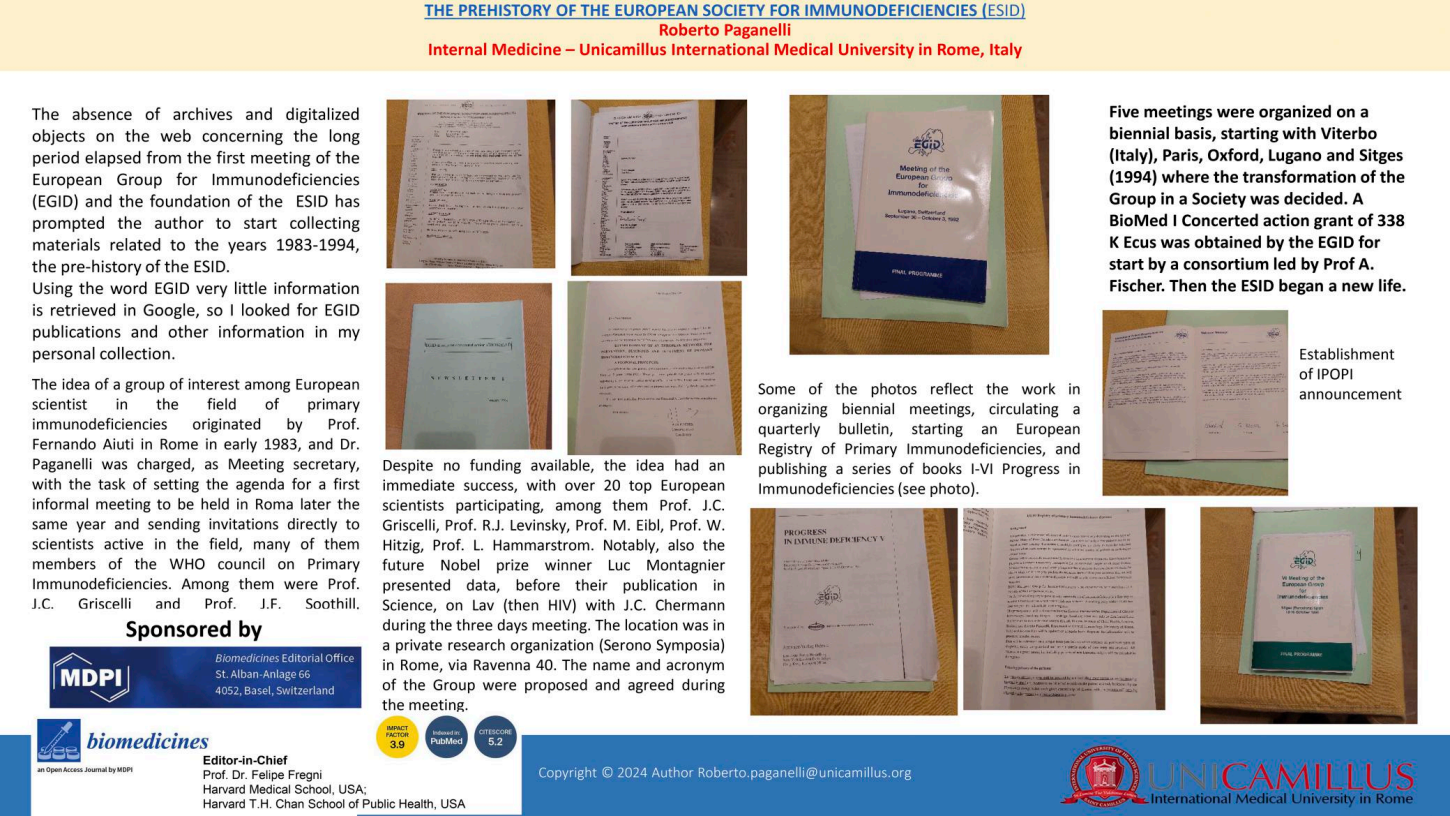
Poster at the 21st biennial meeting of ESID (Marseille, 2024) on the history of EGID.

The path to the success of the ESID-R started even before ESID was created, and many difficulties due to the lack of present technologies were overcome with the dedicated time and efforts of researchers and physicians studying primary immunodeficiencies from all over Europe.

## Online supplemental material

Supplemental material is the reproduction of original documents stored in the author’s personal archive. Fig. S1 shows the front page of the newsletter of the EGID—concerted action (BIOMED-I). Fig. S2 shows a letter from Prof. R. Seger announcing the success of the EGID application to the E.U. Fig. S3 shows a letter from Prof. A. Fischer opening the newsletter of the concerted action. Fig. S4 shows goals and tasks of the EGID project with “1: To build a registry.” Fig. S5 shows a poster at the 21st biennial meeting of ESID (Marseille, 2024) on the history of EGID.

## References

[1] Kindle, G., M. Alligon, M.H. Albert, M. Buckland, J.D. Edgar, B. Gathmann, S. Ghosh, A. Gkantaras, A. Nieters, C. Pignata, . 2025. Inborn errors of immunity: Manifestation, treatment, and outcome–an ESID registry 1994–2024 report on 30,628 patients. J. Hum. Immun. 1:e20250007. 10.70962/jhi.20250007 41347188 PMC12674179

[2] Aiuti, A., R. D’Amelio, I. Quinti, and P. Rossi. 2023. Editorial to the special issue “Clinical immunology in Italy, with special emphasis to primary and acquired immunodeficiencies: A commemorative issue in honor of Prof. Fernando Aiuti”. Biomedicines. 11:3191. 10.3390/biomedicines11123191 38137412 PMC10741147

[3] Fasth, A. 1982. Primary immunodeficiency disorders in Sweden: Cases among children, 1974-1979. J. Clin. Immunol. 2:86–92. 10.1007/BF00916891 6978347

[4] Luzi, G., L. Businco, and F. Aiuti. 1983. Primary immunodeficiency syndromes in Italy: A report of the national register in children and adults. J. Clin. Immunol. 3:316–320. 10.1007/BF00915792 6655036

[5] Rosen, F.S., R.J. Wedgwood, F. Aiuti, M.D. Cooper, R.A. Good, L.A. Hanson, W.H. Hitzig, S. Matsumoto, M. Seligmann, J.F. Soothill, and T.A. Waldmann, 1983. Primary immunodeficiency diseases. Report prepared for the WHO by a scientific group on immunodeficiency. Clin. Immunol. Immunopathol. 28:450–475. 10.1016/0090-1229(83)90112-5 6349887

[6] Abedi, M.R., G. Morgan, H. Goii, R. Paganelli, N. Matamoros, and L. Hammarström. 1998. Report from the ESID registry of primary immunodeficiencies. Mol. Immunol. 35:645–647. 10.1016/S0161-5890(98)90342-X

[7] Abedi, M., G. Morgan, H. Goii, R. Paganelli, N. Matamoros, and L. Hammarström. 2003. Report from the ESID registry of primary immunodeficiencies. The Source. February/March:8–9.

[8] Knerr, V., B. Gathmann, A.M. Eades-Perner, G. Kindle, D. Vett, D. Guzman, and B. Grimbacher. 2005. The ESID online clinical and research database. Centr Eur. J. Immunol. 30:99–103.

[9] ESID Registry Working Party . 2007. The European internet-based patient and research database for primary immunodeficiencies: Results 2004-06. Clin. Exp. Immunol. 147:306–312. 10.1111/j.1365-2249.2006.03292.x 17223972 PMC1810463

